# Scoping review: occupational therapy interventions in primary care

**DOI:** 10.1017/S146342361800049X

**Published:** 2019-03-20

**Authors:** Marije Bolt, Tiska Ikking, Rosa Baaijen, Stephanie Saenger

**Affiliations:** 1 Occupational Therapist, Cordaan – Primary Care Team, Amsterdam, The Netherlands; 2 Occupational Therapist, Evean – Primary Care Team, Wormerveer, The Netherlands; 3 Occupational Therapist, Vermoeidheid and PijnCentrum – Lelystad, The Netherlands; 4 COTEC President, Occupational Therapist, Rolmaat – Abcoude, The Netherlands

**Keywords:** evidence-based practices, home based therapy, interventions, occupational therapy, primary care

## Abstract

This is the second article in a series of two about occupational therapy and primary care. The first article (see *PH&RD*….) described the position of the profession in primary care across Europe and the scope of the profession. In this article the broad scope of the profession is illustrated with various examples of occupational therapy interventions. The interventions are identified by means of a literature search. A questionnaire (the questionnaire is available by mailing the author) was sent out to experts across Europe which resulted in both relevant literature and evidence-based examples. The evidence level of these examples differs from expert opinion (5), case series (4), case–controlled studies (3), cohort studies (2) and randomized-control trial (1). The article ends with recommendations in four areas how to develop, establish or strengthen the profession in primary care.

## Purpose

The purpose of this article is twofold:

To show policymakers, other (healthcare) professionals and managers the variety of the contribution of occupational therapy in primary care,

To support occupational therapists by showing them possible interventions to introduce the profession in primary care, but also to show already established occupational therapists in primary care in which areas they can specialize or start new initiatives.

## Introduction

The project group conducted an initial literature search on occupational therapy interventions being used in Europe in primary care. The project group looked for clinical guidelines, systematic reviews, clinical studies and expert opinions. To identify the studies of interest, a search from inception of the database to March 2017 was conducted. A search for guidelines on the Guidelines International Network with ‘occupational therapy’ as search term resulted in two relevant guidelines about occupational therapy practice guidelines for productive aging for community-dwelling older adults and occupational therapy and physical activity interventions to promote the mental well-being of older people in primary care and residential care, both from the American Occupational Therapy Association. A search for systematic reviews on The Cochrane Library with the search term ‘occupational therapy’ ‘primary care’ resulted in three relevant articles. Furthermore, experts were contacted to detect relevant articles and articles were suggested by participants who provided the project group with feedback.

The following occupational therapy interventions were identified and summarized.

## Interventions

### Assessment and re-training of skills and activities

Observe, assess and evaluate the performance of activities of daily living (ADL), that is, self-care, productivity and leisure activities. Negotiate individual meaningful goals with clients, their caregivers and family and promote shared decision making. Re-train the necessary functional skills and coach the client and his caregivers to achieve optimal independent daily functioning and use his or her full potential.

### Educate on symptom management and manage health conditions

Provide individual or group interventions to help people with mental or physical health issues cope with their condition within the context of their daily lives. For example, chronic pain, chronic fatigue syndrome, diabetes, cognitive impairment, panic disorder, dementia. Inform older adults about health education programs, including self-management programs, which may help lessen pain, improve physical activity levels, and improve participation and functioning (Flannery and Barry, 2003; Arbesman and Mosley, 2012).

### Adapt activities and assistive technology

Inform and provide advice on the use of strategies, techniques and equipment to help people meet their goals. Assist the client to apply for those devices that are reimbursed by the healthcare, municipality or otherwise and train the client in the use of the device. For example, rollator/walker, helping hand, stocking pull, apps for alerting, e-health.

Use client-centred intervention plans that include a ‘mix of exercise, education, home modifications or assistive technology [to provide] the best results in fall prevention and performance support for community-dwelling older adults’ (Chase *et al*., 2012). Consider alternative funding sources to help pay for preventive and health promotion services or equipment not currently reimbursable under medical or private insurance (Stav *et al*., 2012).

### Adapt the social and physical environment

Assess the environmental context in which the client’s everyday activities take place, including housing, workplace, school and socio-cultural environments. Facilitate changes in the environment with the use of assistive technology and welfare technology, rebuilding, task sharing with carers/client system. For example, home safety assessment and modification (falls prevention), arrange help from volunteers, telephone circle, buddy systems, support groups.

### Support caregivers and family

Inform the caregiver about the disease or dysfunction of the client. Work in partnership with the caregiver and identify issues of dis-balance, find alternative ways and solutions and help create a new balance. For example, EDOMAH – occupational therapy for elderly with dementia and their caregivers (also known as COTiD), instructions for transfers, emotional support groups.

## More specialist interventions (College of Occupational Therapists Ltd., 2015)

In some countries occupational therapists are specialized in specific areas of daily living, depending on cultural and environmental circumstances:

- Vocational rehabilitation and reintegration
  Provide specialist input to help people to stay in or return to work (Lambeek *et al*., 2010).
- Fitness to drive
  Assess fitness to drive and/or enable individuals to continue to drive. Integrate diverse driving interventions to positively affect the driving performance of community-dwelling older adults (Orellano *et al*., 2012).
- Lifestyle coaching
  At an individual and community level, addressing a range of health issues by incorporating ‘healthy lifestyle choices’ in daily routines, including the balance between activity, rest and sleep.
- Promoting social inclusion/community engagement
  Provide input at an individual, group or community level to promote social inclusion/community engagement for people at risk of isolation.
  Encourage community-dwelling older adults’ involvement or participation in a variety of occupations and activities to support their health, such as physical activity, social activity, leisure activity, religious activity, work/volunteer work, general activity and instrumental activities daily living (Stav *et al*., 2012).
- Advising technical and adaptive devices
  Assessing and advising the need of (home) care, technical aids, adaptive devices commissioned by local council or insurance companies
- Dance programme
  Six-week dance programme for community-dwelling adults was examined in terms of activity participation, falls efficacy and quality of life. Outcome measures were completed before and after the programme. Results revealed the programme may increase activity participation in social and community-based activities (O’Toole *et al*., 2015).

## Evidence-based practices

The project group sent out a letter to experts, to the members of the primary care group of the COTEC Register of Experts and to other key persons. The letter included a questionnaire to identify projects or occupational therapy interventions and research findings which support the positive contribution of occupational therapy in primary care.

There is strong evidence that occupational therapy improves functioning in community-dwelling physically frail older people (De Coninck *et al*., 2017). Other studies provide evidence of the positive cost-effectiveness of a specific occupational therapy intervention. For example the COTiD-research (Graff *et al*., 2007; Graff *et al*., 2008; University College London, 2017) and the OTiP study (Sturkenboom *et al*., 2014).

A total of 11 examples of best practices were given. The evidence level of these examples differs from expert opinion (5), case series (4), case–controlled studies (3), cohort studies (2) and randomized-control trial (1).

### Community-based occupational therapy for older people with dementia and their caregivers (COTiD) (Graff *et al*., 2007; 2008): The Netherlands (level 1)

In this study randomised controlled trial (RCT) it was found that a community-based occupational therapy intervention (two occupational therapy interventions of 1 h a week for five weeks) had positive effects on the quality of life, mood and health status in dementia clients and their caregivers. The intervention was very cost-effective. This intervention is also being implemented and researched in Italy, France and the United Kingdom (University College London, 2017). Germany and Switzerland will follow in the near future.

### Occupational therapy for people with Parkinson’s disease (OTiP) (Sturkenboom *et al*., 2014): The Netherlands (level 1)

In this study RCT it was shown a home-based individualized occupational therapy intervention led to an improvement in self-perceived performance in daily activities in persons with Parkinson disease. Although occupational therapy did not significantly impact on total costs compared with usual care, positive cost-effectiveness of the intervention was significant for caregivers.

### OPTIMAL (Garvey *et al*., 2015): Ireland (level 1)

OPTIMAL is a six-week community-based programme, led by occupational therapy facilitators and focuses on problems associated with managing multi-morbidity. The primary outcome was frequency of activity participation. Secondary outcomes included self-perception of, satisfaction with and ability to perform daily activities, independence in ADL, anxiety and depression, self-efficacy, health-related quality of life, self-management support, healthcare utilization and individualized goal attainment. OPTIMAL significantly improved frequency of activity participation, self-efficacy and quality of life for clients with multi-morbidity.

### Reablement (Kjerstad and Tuntland, 2016; Langeland *et al*., 2016; Liaaen and Vik, 2016): Norway (levels 1 and 2)

In Norway the intervention ‘reablement’ has been thoroughly investigated. Re-ablement means a form of home-based rehabilitation, which focuses on improving independent functioning in daily activities perceived as important by the older adult.

In a Norwegian study Reablement was found to be more cost-effective than usual care. The assessments of performance and satisfaction regarding daily activities were significantly higher in the reablement group compared with the control group and this was achieved at lower cost. In the post-trial period, the intervention group requested significantly fewer home visits which were, on average, of significantly shorter duration compared with the control group. Expenditure on home visits was significantly lower for the reablement group.

### Cooperation with homecare staff (Hjelle *et al*., 2016; Langeland *et al*., 2016): Norway (level 5)

A Norwegian study investigated how structured teaching in daily life activities and everyday coping can improve cooperation between the occupational therapist and community-based homecare staff. Results showed that structured training can improve the collaboration between occupational therapists and the homecare staff. There is a need to work thoroughly and continuously in order to implement new methods and work with attitude changes in order to achieve a common platform and increase multidisciplinary collaboration in community services.

### Men’s Health Promotion Group (Tinnelly *et al*., 2014): Ireland (level 4)

Communicating and interacting with men in familiar environments is thought to be essential for engaging with men. This eight week occupational therapy group aimed to encourage men to become active agents and self-advocates for their own health, provide an environment to engage men in discussing health-related issues and concerns, explore community resources and health-related services available to men, and examine the effects of a group-based programme on client’s quality of life, well-being and engagement with the community. Peer support, healthy ageing focus and improved awareness of health and health services are central to this occupation-focused group.

### Memory and lifestyle (Tinnelly *et al*., 2016): Ireland (level 4)

An immediate need to intervene with a population health approach to dementia prevention has been identified. Primary health promotion interventions are suitable methods of addressing modifiable risk factors and augmenting protective factors in primary care. A four-week community-based occupational therapy intervention was developed grounded in this knowledge. The intervention adopted an upstream approach, addressing cognitive complaints and lifestyle factors in order to promote healthy ageing and brain health. Important improvements for participants were noted. In particular these related to increased satisfaction relating to occupational performance has positive implications for continued engagement in meaningful activities and promoting successful ageing.

### Child and family (Bargao Rodrigues, 2016): Portugal (level 5)

Early Intervention Teams Locations in Childhood (ELI Alcanena/Torres Novas) under the National Service for Early Childhood Intervention (SNIPI) of the Portuguese government offer a set of integrated support measures focused on the child and family, including preventive and rehabilitative actions in education, health and social areas. The occupational therapist in the Health Centre also responds to requests from GP’s, particularly in domiciliary intervention, child and school health and awareness raising in the community, among others.

### Adults after cerebrovascular accident [(CVA) Scheffer, 2016]: The Netherlands (level 5)

Integrated/client-centred and multi-professional care for people after a stroke/CVA, in the acute, post-acute period and also the chronic period after stroke. The easily accessible care at home, the collaboration with a primary care based neuro-psychologist and a multidisciplinary team based on the clients’ needs, are the success factors of this intervention.

### Adults with attention-deficit hyperactivity disorder (ADHD) and [autistic-spectrum disorder (ASD) Lamers, 2017]: The Netherlands (level 5)

The Skype prompting group supports adults with ADHD and ASD who suffer from procrastination to get their tasks done. The occupational therapist meets with three to five procrastinators on Skype each week on a regular time, which supports the participants to get different types of chores done and boosts their self-esteem and self-confidence.

### Community care service (Adamina Swante Gerritsma, 2016): Spain (level 5)

A pro-active model was organized within the municipal Social Services in the Barcelona region in which the occupational therapist facilitates and coordinates various services to the individual, caregiver and community. The basic characteristics of the project focus on person-centred and integrated care. The occupational therapist functions as a bridge between the municipality’s Social Services and public or private, physical or mental health and community care services.

### Health promotion (Tinnelly, 2016): Ireland (level 5)

There are some very valuable examples of health promotion interventions in Ireland including Men’s Health Promotion Group, Stress and Wellbeing Group, Memory and Lifestyle Group (for Mild Cognitive Impairment) and Falls Prevention Group.

### Oncology (HOPE, 2004; World Health Organization, 2016): UK, The Netherlands (level 5)

People with cancer benefit from occupational therapy assessment and intervention across their lifespan and disease. Occupational therapists provide assessment, intervention and support during, between and after active treatment and, if necessary, care at the end of life. Key elements are lifestyle management, fatigue management and enhancing self-esteem. Symptoms, for example pain, fatigue and breathlessness, influence activities. Occupational therapy intervention is aimed to reduce the burden of the symptoms on the activity and to increase quality of life.

### Mental health and chronic pain (World Health Organization, 2016): The Netherlands (level 5)

Occupational therapy at home for people with mental health problems and chronic pain improves independent functioning, self-esteem and creates more acceptances from the social environment. Success factors are the client centered approach, paced and concrete situations and the eclectic treatment. Several measurements are useful, for example the Activity Balancer (Hove and Hulstein, 2017) and the Role Checklist (Kielhofner, 2002).

### Mental health and supported employment (Nugteren, 2017): The Netherlands (level 5)

occupational therapy/therapist (OT) restores involvement in work. An OT combines an individual training, focused on increasing self-esteem, expressing feelings and assertiveness with graded activity training, psycho-education (both client and colleagues), training on the job and the necessary adaptations of the workplace. The other disciplines involved are a physiotherapist, social legal worker and the employer.

## Recommendations

Based on the findings of the questionnaire, the opinion of experts, the best practices and the feedback out of discussions with occupational therapists during the OT-EU workshop (Ikking et al., 2016), the following recommendations were identified. The nature of the recommendations varies and will not apply in every situation, region or country. However, the recommendations are clearly described/formulated on the following aspects:

- Focus on occupational therapy services (specialist versus generalist) and professional development
- Making alliances
- Public relations
- Financing and accessibility.

Recommendations should be executed on macro-, meso- and micro-level, by governments, policymakers, (inter) national occupational therapy associations and individual occupational therapists. Some recommendations overlap.

## (A) Focus on occupational therapy services and professional development

1. Make a clear choice between generalists and specialists occupational therapy services;

- offer a broad scope of interventions to many target groups;
- or focus on one or two target groups and/or specialist interventions.

To offer a broad scope of interventions to many targets groups can be positive as it shows the many possibilities of the occupational therapy profession, but in the first phase of developing the profession in primary care it is recommended to focus on one or two target groups (based on, eg, context needs, financing, alliances, research findings, expertise of OT’s involved). In the next phase, when occupational therapy is more established and known, more target groups and/or interventions can be developed.

Be aware of minority groups in your society and establish collaboration with minority groups that you do not normally work with. Mediation has proved to be an effective tool. Skills training of primary care practitioners may enhance their individual competences (De Graaf *et al*., 2017).

2. Professional development

- Work together with occupational therapy education
  - The level of occupational therapy education in Europe differs. Therefore, not all recently graduated occupational therapists are seen as capable of working in primary care. Creating a uniform educational level of the profession across Europe will enhance the capacity of occupational therapists to work within primary care.
  - An occupational therapist in primary care needs many different competences, the following competences are rated very high
    - interdisciplinary work
    - goal-oriented work
    - client-centred approach
    - shared decision making
    - entrepreneurial skills
  - Establishing occupational therapy in primary care can be facilitated by educational projects, for example fieldwork in new areas within the community and student research projects
- Organize training in entrepreneurial skills and PR for occupational therapists on individual level
- Organize Continuous Professional Development courses for occupational therapists targeted on specific client groups in Primary Care
- Conduct cost-effectiveness studies regionally or locally to provide evidence occupational therapy in primary care is cost-effective

## (B) Making alliances

1. On local, regional and/or national level with non-occupational therapists:

- Specific primary care professionals for example general practitioner’s/nurse practitioners, community nurses and other allied health and social professionals
- The client and consumer groups
- The community health centres
- Social services
- Hospitals and rehabilitation centres
- Schools and other educational institutes
- Health and social insurance companies
- Research institutes
- European level: COTEC and OT-EU are advised to collaborate and make or strengthen alliances with European Forum for Primary Care (EFPC), WHO-EU region like in CIHSD and Mental Health consortium, European Patients Forum (EPF), Eurocarers, European Network on Patient Empowerment (ENOPE), International Foundation for Integrated Care (IFIC).
  2. On local, regional and/or national level with occupational therapists
- In institutional care and specifically in primary care;
- With the (few) occupational therapists that already work in private practice in your country or when there are many organize yourself in an interest/expert/inter-vision group (eg, as part of the national association);
- Individuals, national OT associations and their specialists’ sections in other countries.

## (C) Public relations

It is important to promote occupational therapy at micro-, meso- and macro-level. Be sure to emphasize occupational therapists can work in health (physical and mental) and social areas.

- Identify the local needs and develop a tailored occupational therapy intervention
- Identify the possibilities for financing
- Identify the key stakeholders and politicians
- Know and use facts, figures, evidence and examples of your country or region
- Know and use facts, figures, evidence and examples of related regions or countries as inspiration
- Develop PR material and develop PR campaigns targeted on specific groups like
  - referrers
  - the clients
  - the general public
  - policymakers
  - insurance companies
  - other stakeholders
  to enhance the knowledge of occupational therapy. The use of client’s story has been proved to be effective.
- Promoting occupational therapy in primary care can be facilitated by educational projects, for example fieldwork in new areas within the community and student research projects

## (D) Financing and accessibility

To increase occupational therapy in primary care it is important to find ways of financing. As systems, laws and regulations in countries and regions in Europe differ, it is impossible to give an overview or specific recommendations.

- It is essential to investigate all regular and alternative ways of financing
  - Being creative and entrepreneurial is essential for success
- Referral systems often are linked with financing
  - Accessibility to OT services without referral (direct access and private paying) seems in many countries the only way
  - Accessibility to occupational therapy without referral improves patient autonomy and empowerment
  - Pilot Projects with client groups and insurance can provide positive results for further financing
  - Cost-effectiveness studies can provide evidence occupational therapy is cost-effective, make use of (inter) national conducted studies

## Conclusions

The major contribution of occupational therapy seems to be the client-centred, holistic and goal-oriented approach. Especially the occupational perspective focussed on the enablement of daily living and participation of individuals of all ages, their caregivers, groups and communities in society is recognized as being worthwhile.

For special groups (eg, dementia, stroke, Parkinson disease) studies show that occupational therapy interventions increase the quality of life, improve the self-perceived performance of daily activities for clients and their caregivers and are cost-effective.

The recommendations show many actions can be taken on many levels to improve the position of Occupational Therapy in Primary Care.

To collaborate with other professionals, local and regional policymakers and with the educational institutes for occupational therapy is essential to show the relevance of the contribution of the profession in primary care as is the need for further (contextual) research on interventions.

## Discussion

The project group is very much aware of the limitations of this article. First of all, the members of the Project Group, although with a European knowledge and experience, are based in the Netherlands. Secondly, most of the content is based on information given by others and not all COTEC members, nor experts have answered the survey and/or the questionnaire.

Knowing the great differences in Europe and wanting to capture as many examples as possible the survey had a very broad scope and did not ask for any details like theories or frames of reference behind the interventions nor for specific assessments used in primary care in Europe. When finalizing the article the project group got acquainted with the work of Starfield (Macinko *et al*., 2003). Starfield (1994) approaches primary care by defining 10 components which together determine the concept of primary care. These components are regulation, financing, type of primary care physician, access, longitudinality, first contact, comprehensiveness, family-centeredness and community orientation. Based among others on Starfield’s work it can be expected that countries in Europe vary in the strength of key features of primary care. It would be worthwhile to repeat the questionnaire with the focus on these components.

The project group is confident in the near future more research will be conducted and the number of evidence-based practices will grow.

Despite these limitations, the authors hope that this article will serve as inspiration for further research, study and policy making.
